# IGF-1/IGF-1R/*hsa-let-7c* axis regulates the committed differentiation of stem cells from apical papilla

**DOI:** 10.1038/srep36922

**Published:** 2016-11-11

**Authors:** Shu Ma, Genxia Liu, Lin Jin, Xiyao Pang, Yanqiu Wang, Zilu Wang, Yan Yu, Jinhua Yu

**Affiliations:** 1 Guiyang Hospital of Stomatology, 253 Jiefang Road, Guiyang, Guizhou 550002, China; 2 Key Laboratory of Oral Diseases of Jiangsu Province and Stomatological Institute of Nanjing Medical University, 140 Hanzhong Road, Nanjing, Jiangsu 210029, China; 3 Endodontic Department, School of Stomatology, Nanjing Medical University, 136 Hanzhong Road, Nanjing, Jiangsu 210029, China

## Abstract

Insulin-like growth factor-1 (IGF-1) and its receptor IGF-1R play a paramount role in tooth/bone formation while *hsa-let-7c* actively participates in the osteogenic differentiation of mesenchymal stem cells. However, the interaction between IGF-1/IGF-1R and *hsa-let-7c* on the committed differentiation of stem cells from apical papilla (SCAPs) remains unclear. In this study, human SCAPs were isolated and treated with IGF-1 and *hsa-let-7c* over/low-expression viruses. The odonto/osteogenic differentiation of these stem cells and the involvement of mitogen-activated protein kinase (MAPK) pathway were subsequently investigated. Alizarin red staining showed that *hsa-let-7c* low-expression can significantly promote the mineralization of IGF-1 treated SCAPs, while *hsa-let-7c* over-expression can decrease the calcium deposition of IGF-1 treated SCAPs. Western blot assay and real-time reverse transcription polymerase chain reaction further demonstrated that the expression of odonto/osteogenic markers (ALP, RUNX2/*RUNX2*, OSX/*OSX*, OCN/*OCN*, COL-I/*COL-I*, DSPP/*DSP*, and DMP-1/*DMP-1*) in IGF-1 treated SCAPs were significantly upregulated in *Let-7c*-low group. On the contrary, *hsa-let-7c* over-expression could downregulate the expression of these odonto/osteogenic markers. Moreover, western blot assay showed that the JNK and p38 MAPK signaling pathways were activated in *Let-7c*-low SCAPs but inhibited in *Let-7c*-over SCAPs. Together, the IGF-1/IGF-1R/*hsa-let-7c* axis can control the odonto/osteogenic differentiation of IGF-1-treated SCAPs via the regulation of JNK and p38 MAPK signaling pathways.

Mesenchymal stem cells (MSCs) are multipotent progenitor cells, originally isolated from the adult bone marrow or other issues, which can differentiate into many kinds of different cell types^1^. Various growth factors can mediate the osteo/dentinogenic differentiation of MSCs^2^,^3^,^4^,^5^, including stem cells from apical papilla (SCAPs)^6^. These SCAPs can differentiate into odontoblasts or osteoblasts *in vitro*, and form dentin-like tissues along with tooth root-like structures *in vivo*^1^,^3^,^7^.

Insulin-like growth factor (IGF) axis plays a paramount role in bone formation, maintenance of mineralized skeletons as well as the process of tooth formation. One of the polypeptide growth factors (i.e., IGF-1) is the most important member of this axis^7^. Previous studies have revealed that IGF-1 can significantly enhance the odonto/osteogenic differentiation of SCAPs, periodontal ligament stem cells (PDLSCs) and dental pulp stem cells (DPSCs)^6^,^8^,^9^. IGF-1 receptor (IGF-1R) is a cell membrane receptor that contains two transmembrane α subunits and two cytosolic β subunits covalently bonded through disulphide bridges^7^. It is commonly believed that IGF-1R can respond to IGF-1 to stimulate the osteoblast proliferation and bone matrix mineralization^10^,^11^. Moreover, IGF-1 can regulate the cell differentiation by activating the downstream signaling pathways through its receptor IGF-1R, and IGF-1/IGF-1R pathway is thereby known as IGF-1/IGF-1R axis.

MicroRNAs (miRNAs) are small RNA molecules and comprised of 21–25 nucleotides that modulate the inhibition of translation from mRNA to protein, promotion of mRNA degradation and control of gene transcription via binding to the 3′-untranslated region (UTR) of their target mRNAs^12^. The *let-7* family of miRNAs is initially detected in caenorhabditis elegans and identified as a heterochronic gene, which is considered as a miRNA biomarker in osteoblastic and osteocytic differentiation of mesencymal stem cells^13^,^14^. Recent studies have demonstrated that the downregulation of *let-7* can upregulate the expression of IGF1/IGF1R by mediating the IGF1 signaling cascade to promote the differentiation of spermatogonia into primary spermatocytes^15^. It has been reported that the overexpression of *hsa-let-7c* can downregulate the renewal capacity of stem cells subsets, and the upregulation of expression of *hsa-let-7c* is related with the mechanism of GH/IGF-1 axis signaling pathway^16^,^17^.

To date, the effects of IGF-1/IGF-1R/*let-7c* axis as a whole on the odonto/osteogenic differentiation of SCAPs and its underling mechanism remain unclear. In this study, we investigated the influence of IGF-1/IGF-1R/*hsa-let-7c* axis on the proliferation and differentiation of human SCAPs, and found that IGF-1/IGF-1R/*hsa-let-7c* axis can affect the odonto/osteogenic differentiation of SCAPs during which JNK and p38 MAPK signaling pathway were involved. The accumulating findings may implicate a novel approach to dental tissue regeneration as well as apexogenesis.

## Results

### Identification of SCAPs and screening of MOI values for lentiviral infection

**Figure 1:**
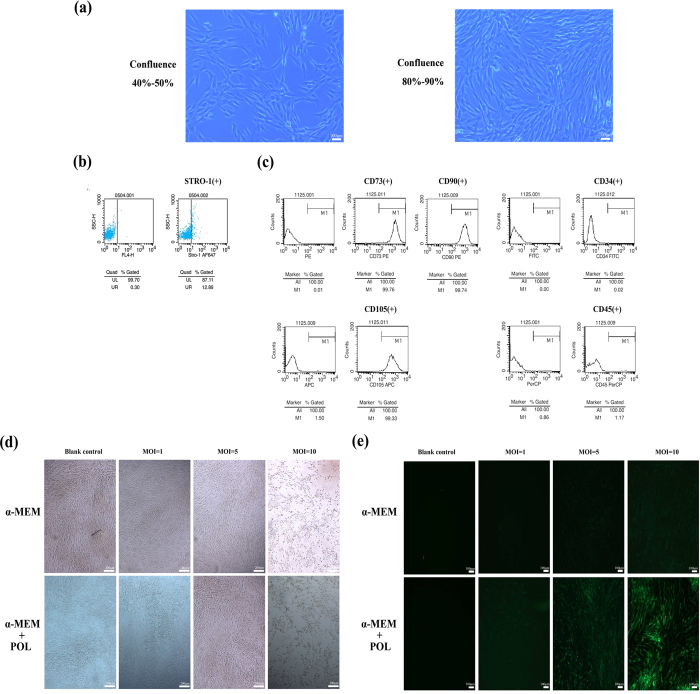
Identification of SCAPs and screening of MOI values for lentiviral infection. **(a)** Isolated SCAPs with typical fibroblast- or spindle-like morphology. **(b)** Isolated SCAPs were immunopositive against STRO-1 by flow cytometry. **(c)** Isolated SCAPs were positive for CD73, CD90 and CD105, respectively, but negative for CD45 and CD34 by flow cytometry. **(d)** Cell morphology under microscope in α-MEM and α-MEM+POL (Polybrene) groups with different virus titers. **(e)** Cell fluorescence expression under an inverted microscope in α-MEM and α-MEM+POL groups with different virus titers.

The isolated SCAPs presented a typical fibroblast- or spindle-like morphology under microscope at 40–50% and 80–90% confluence respectively (Fig. 1a). FCM results revealed that the isolated cells were immune-positive against STRO-1 (a mesenchymal stem cell marker, Fig. 1b). Moreover, CD73, CD90 and CD105 were highly expressed, while CD45 and CD34 were lowly expressed in these cells, indicating that these isolated cells were stem cells of mesenchymal origin (Fig. 1c) without contamination of hematopoietic precursors.

With the gradual increase of MOI values, the toxic effects of the virus on SCAPs increased too. SCAPs in conventional medium (α-MEM) groups and conventional medium plus polybrene (POL) groups became elongated as compared with the blank control under the microscope (Fig. 1d). The fluorescence expression of conventional medium groups was weaker than conventional medium plus POL groups as compared with the negative control under an inverted microscope. When the virus titer is 5 × 10^6^ TU/mL, the cell morphology and fluorescence expression in conventional medium + POL group were better than other groups (Fig. 1e). The best MOI value equals to 5 via the calculation of fluorescence microscope images as well as the evaluation of cell vitality (Fig. 1d,e).

### IGF-1R protein expression is negatively correlated with *let-7c* expression

**Figure 2:**
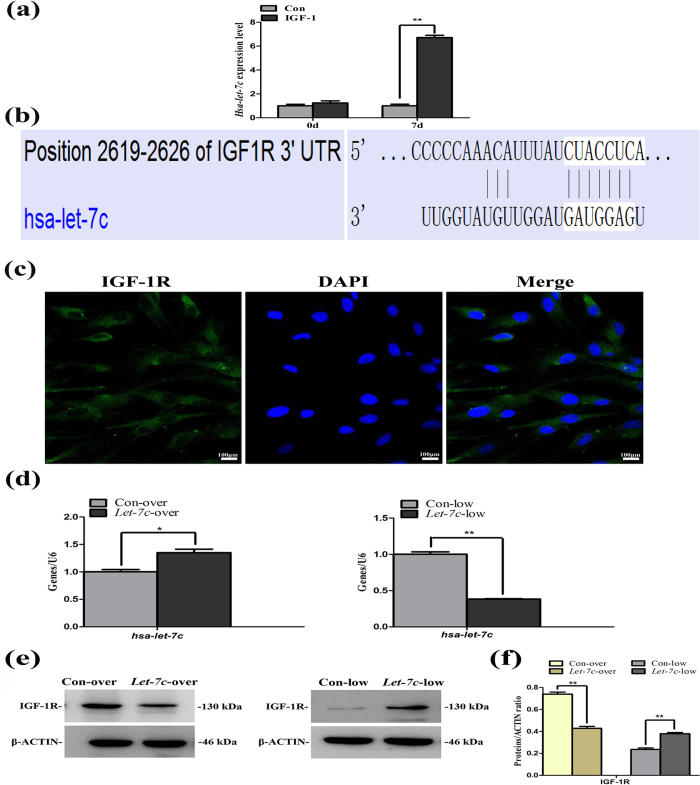
Negative correlation between *hsa-let-7c* and IGF-1R. **(a)** *Hsa-let-7c* detection in IGF-1-treated SCAPs by miRNA microarray. **(b)** The predicted consequential pairing of target region (top) and miRNA (bottom) between *IGF-1R* and *hsa-let-7c* on the TargetScanHuman website. **(c)** Immunofluorescent staining of IGF-1R in SCAPs. Nuclei are stained in blue and IGF-1 receptor was in green. **(d)** Real-time RT-PCR analysis for the expression of *hsa-let-7c* in Con-over group, *Let-7c*-over group, Con-low group and *Let-7c*-low group, respectively. Values were described as the means ± SD, n = 3. **2^−∆∆Ct^ > 2, *P* < 0.01; *1 < 2^−∆∆Ct^ < 2, *P* < 0.01. **(e)** Western blot assay for the expression of IGF-1R in Con-over group, *Let-7c*-over group, Con-low group and *Let-7c*-low group, respectively. **(f)** Quantitative analysis for western blot results. Values were described as the means ± SD, n = 3. **P < 0.01.

We utilized the miRDB database bioinformational analysis and found that IGF-1R was one member of the target genes of *hsa-let-7c* with a target score up to 91 points. Then we verified the target gene of *hsa-let-7c* through the gene and protein detection. We performed the miRNA microarray to detect the related miRNA levels in IGF1-treated SCAPs and found that *hsa-let-7c* expression in treated cells increased 6.68 folds as compared with control group (Fig. 2a). Meanwhile, we obtained the sequence of *hsa-let-7c* (UGAGGUAGUAGGUUGUAUGGUU) on the miRBase website. Then we found the predicted consequential pairing of target region (top) and miRNA (bottom) between IGF-1R and *hsa-let-7c* on the TargetScanHuman website (Fig. 2b), confirmed the specific sequence of *hsa-let-7c* (UGAGGUAGUAGGUUGUAUGGUU), and thus acquired the real-time sequence (Table 1).

The immunofluorescent results demonstrated that IGF-1R was mainly located in the cytoplasm of SCAPs (Fig. 2c). Real-time RT-PCR results confirmed that the expression of *hsa-let-7c* increased in *Let-7c* over-expression group (*Let-7c*-over), but decreased in *Let-7c* low-expression group (*Let-7c*-low, Fig. 2d, *P* < 0.01). Western blot revealed that the low expression of *let-7c* could upregulate the expression of IGF-1R, whereas the over-expression of *let-7c* downregulated the expression of IGF-1R (Fig. 2e,f).

### Effects of IGF-1/IGF-1R/*hsa-let-7c* axis on proliferation of SCAPs

**Figure 3:**
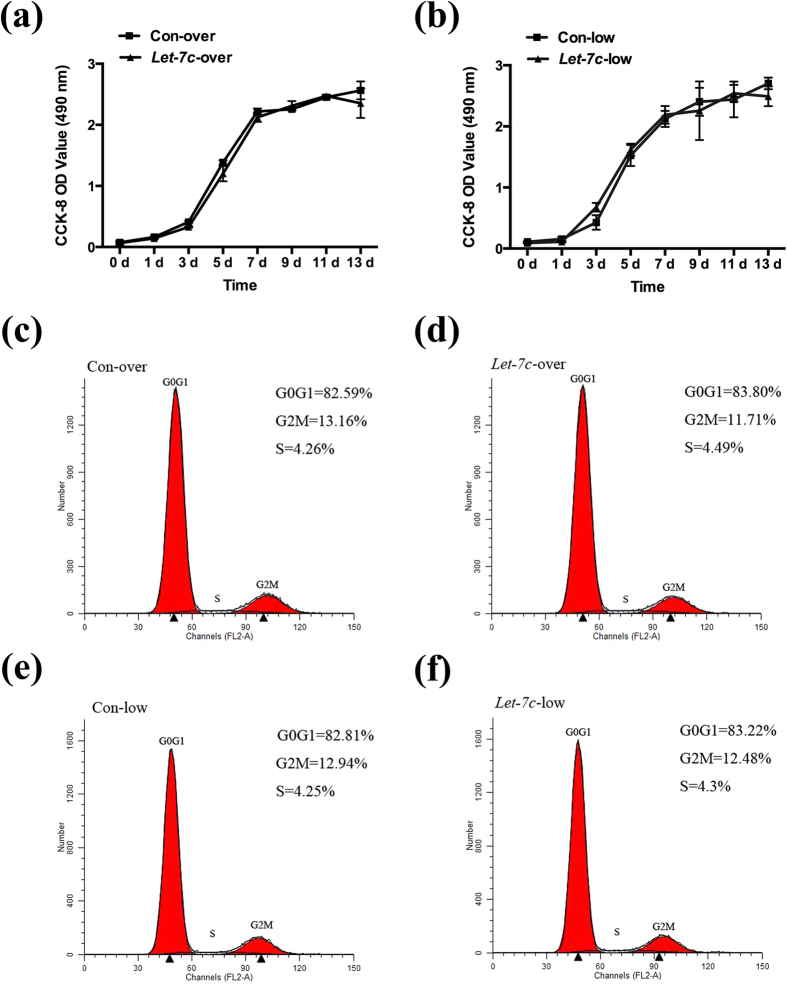
IGF-1/IGF-1R/*hsa-let-7c* axis had no significant influence on the proliferation of SCAPs. **(a)** CCK8 assay of 13 consecutive days presented no significant difference (*P* > 0.05) between Con-over group and *Let-7c*-over group. **(b)** CCK8 assay of 13 consecutive days displayed no significant difference (*P* > 0.05) between Con-low group and *Let-7c*-low group. **(c)** Flow cytometry (FCM) analysis of Con-over group. **(d)** Flow cytometry analysis of *Let-7c*-over group. **(e)** Flow cytometry analysis of Con-low group. **(f)** Flow cytometry analysis of *Let-7c*-low group.

CCK8 assay and FCM were performed to investigate whether the IGF-1/IGF-1R/*hsa-let-7c* axis can affect the proliferation of SCAPs through the over/low-expression of *let-7c*. CCK8 assay on 11 consecutive days presented no significant difference (*P* > 0.05) between two groups (Fig. 3a,b). FCM analysis further showed no distinct difference (*P* > 0.05) in the proliferation index (PI = G_2_M+S) between Con-over group (17.42%) and *Let-7c*-over group (16.2%, Fig. 3c,d). Likewise, FCM analysis demonstrated no distinct difference (*P* > 0.05) in the proliferation index (PI) between Con-low group (17.19%) and *Let-7c-*low group (16.78%, Fig. 3e,f). Together, IGF-1/IGF-1R/*hsa-let-7c* axis had no significant effect on the proliferation of SCAPs.

### Effects of IGF-1/IGF-1R/*hsa-let-7c* axis on odonto/osteogenic differentiation of SCAPs

**Figure 4:**
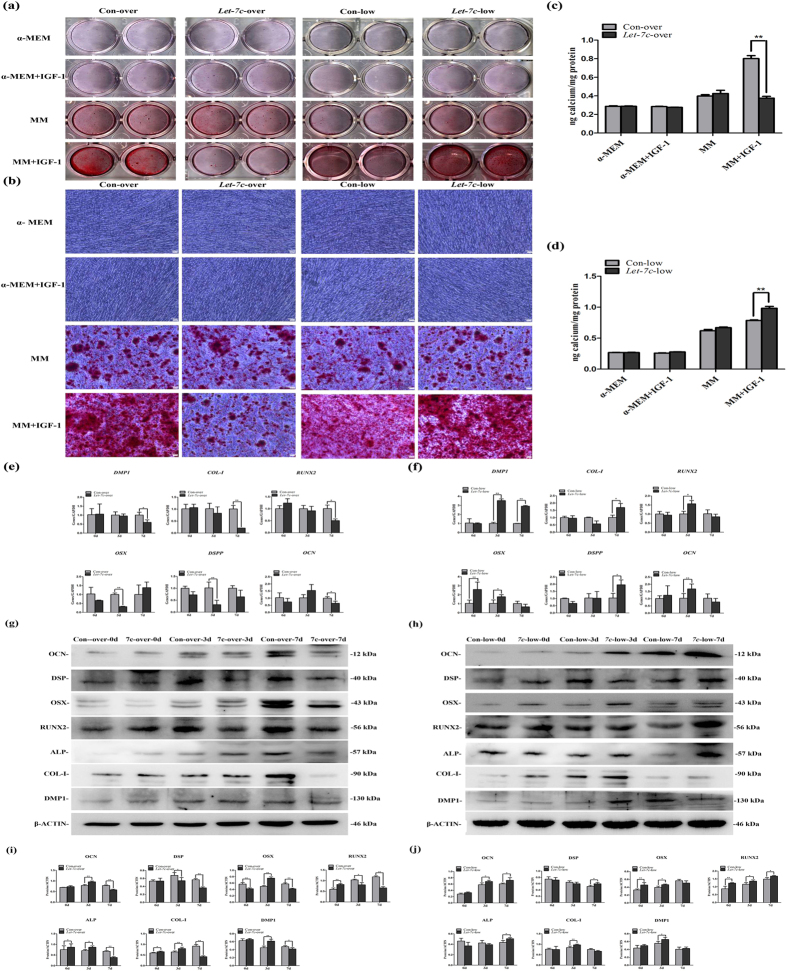
IGF-1/IGF-1R/*hsa-let-7c* axis can regulate the odonto/osteogenic differentiation of SCAPs. **(a)** Alizarin red staining for control group, insulin-like growth factor-1 (IGF-1) group, mineralization-inducing media (MM) group and MM+IGF-1 group at day 14, respectively. **(b)** Calcium nodules in different groups under the inverted microscope. *Scale bars* = 100 μm. **(c)** Quantitative analysis for calcium contents in the *Let-7c*-over group. Values were the means ± SD, n = 3. **P < 0.01. (**d**) Quantitative analysis for calcium contents in the *Let-7c*-low group. Values were the means ± SD, n = 3. **P < 0.01. (**e**) Real-time RT-PCR analysis for the expression of *DMP-1, COL-I, RUNX2, OSX, DSPP* and *OCN* in Con-over group and *Let-7c*-over group respectively. (**f**) Real-time RT-PCR analysis for the expression of *DMP-1, COL-I, RUNX2, OSX, DSPP* and *OCN* in Con-low group and *Let-7c*-low group respectively. Values were described as the means ± SD, n = 3. **2^−∆∆Ct^ > 2, *P* < 0.01; *1 < 2^−∆∆Ct^ < 2, *P* < 0.01. (**g**) Western blot assay for the odonto/osteogenic proteins (OCN, DSP, OSX, RUNX2, ALP, COL-I and DMP1) in Con-over group and *Let-7c*-over group respectively. (**h**) Western blot assay for the odonto/osteogenic proteins (OCN, DSP, OSX, RUNX2, ALP, COL-I and DMP1) in Con-low group and *Let-7c*-low group respectively. (**i**) Quantitative analysis for western blot results in Con-over group and *Let-7c*-over group respectively. Values were described as the means ± SD, n = 3. *P < 0.05, **P < 0.01. (**j**) Quantitative analysis for western blot results in Con-low group and *Let-7c*-low group respectively. Values were described as the means ± SD, n = 3. *P < 0.05, **P < 0.01.

SCAPs were cultured in α-MEM or mineralization media (MM) containing IGF-1 after transfection with *hsa-let-7c* over/low-expression viruses. Alizarin red staining and quantitative calcium measurement demonstrated that SCAPs with the low-expression of *hsa-let-7c* produced more calcium nodules after 14 days of osteogenic induction in MM+IGF-1 group than the control group. However, SCAPs with the over-expression of *hsa-let-7c* generated less calcium nodules after 14 days of induction in MM+IGF-1 group than the control group (Fig. 4a–d, *P* < 0.01).

Real-time RT-PCR findings revealed that the odonto/osteogenic genes including *DMP1, DSPP, RUNX2, OSX* and *OCN* obviously decreased in IGF-1-treated SCAPs accompanying with the *hsa-let-7c* over-expression, in which the expression of *DSPP* and *OSX* reduced at day 3, while *COL-I, RUNX2, OCN, DMP1* reduced at day 7 (Fig. 4e, *P* < 0.05). However, the mRNA levels of these markers were significantly enhanced in IGF-1-treated SCAPs accompanying with the low-expression of *hsa-let-7c*. In particular, the expression of *RUNX2, OSX* and *OCN* was remarkably increased at day 3, and the expression of *COL-I* was raised at day 7, while the expression of *DMP1* was upregulated at day 3 and 7 (Fig. 4f, *P* < 0.05).

Western blot results demonstrated that the expression of the odonto/osteogenic proteins (DMP1, COL-I, ALP, RUNX2, OSX, DSP and OCN) was significantly decreased in IGF-1 treated SCAPs accompanying with the over-expression of *hsa-let-7c*, in which the expression of RUNX2 and DSP kept reducing from day 3 to day 7, and the expression of DMP1, COL-I, ALP, OSX and OCN obviously decreased at day 7 (Fig. 4g,i, *P* < 0.05). In contrast, these protein expressions were significantly upregulated in IGF-1-treated SCAPs accompanying with the low-expression of *hsa-let-7c*. In detail, the expression of DMP1, COL-I, and OSX increased at day 3, while the expression of ALP, RUNX2, and DSP was enhanced at day 7, and the expression of OCN was upregulated at both day 3 and 7 (Fig. 4h,j, *P* < 0.05).

### Effects of IGF-1/IGF-1R/*hsa-let-7c* axis on MAPK pathway in SCAPs

**Figure 5:**
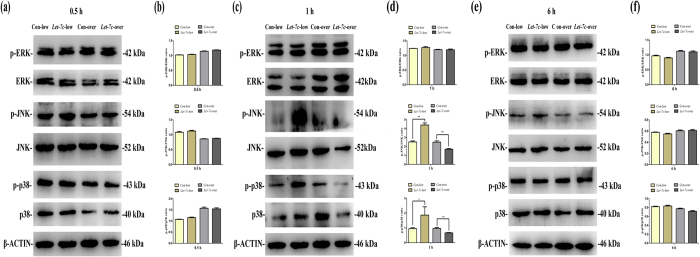
IGF-1/IGF-1R/*hsa-let-7c* axis can regulate MAPK signaling pathway in SCAPs. **(a)** Western blot assay for the expression of MAPK related proteins at 0.5 hour (ERK, phosphorylated ERK, JNK, phosphorylated JNK, p38 and phosphorylated p38, respectively). **(b)** The ratio changes of p-ERK/ERK, p-JNK/JNK and p-p38/p38 at 0.5 hour in different groups. Values were described as the means ± SD, n = 3. *P < 0.05, **P < 0.01. **(c)** Western blot assay for the expression of MAPK related proteins at 1 hour. **(d)** The ratio changes of p-ERK/ERK, p-JNK/JNK and p-p38/p38 at 1 hour in different groups. Values were described as the means ± SD, n = 3. *P < 0.05, **P < 0.01. **(e)** Western blot assay for the expression of MAPK related proteins at 6 hour. **(f)** The ratio changes of p-ERK/ERK, p-JNK/JNK and p-p38/p38 at 6 hour in different groups. Values were described as the means ± SD, n = 3. *P < 0.05, **P < 0.01.

To elucidate the potential involvement of MAPK signaling pathway in IGF-1/*hsa-let-7c* mediated differentiation of SCAPs, we respectively collected the cytoplasmic proteins in Con-over group, *Let-7c*-over group, Con-low group and *Let-7c*-low group and investigated the expression of MAPK related proteins by western blot at 0.5 hour (Fig. 5a,b), 1 hour (Fig. 5c,d) and 6 hour (Fig. 5e,f) respectively. The levels of phosphorylated ERK, phosphorylated JNK and p38 almost did not change at both 0.5 hour and 6 hour (Fig. 5a,e, respectively). Quantitatively, the ratio of p-ERK/ERK, p-JNK/JNK and p-p38/p38 were not affected in both *Let-7c*-low group and *Let-7c*-over group (Fig. 5b,f, respectively). As shown in Fig. 5c (1 hour), the level of phosphorylated ERK almost did not change. However, the levels of phosphorylated JNK and p38 were significantly enhanced in *Let-7c*-low group, as compared with *Let-7c*-over group (Fig. 5c). Quantitatively, the ratio of p-ERK/ERK was not affected in both *Let-7c*-low group and *Let-7c*-over group (Fig. 5d). However, the ratios of p-JNK/JNK and p-p38/p38 noticeably increased at 1 hour in *Let-7c*-low group, while the ratios of p-JNK/JNK and p-p38/p38 remarkably decreased at 1 hour in *Let-7c*-over group (Fig. 5d, *P* < 0.05 or *P* < 0.01).

## Discussion

The *let-7* family which consists of several members (*let-7a/b/c/d/e/f/g/i*) plays a crucial role in various cellular activities including the committed differentiation of multiple cell types^14^,^18^,^19^. All different *let-7* family members have the common seed motif thereby likely sharing the same target genes^15^, in which IGF-1R is identified as a target gene of *let-7* miRNAs^15^. Magnucki *et al*. have detected that IGF-1R is expressed in both DPSCs and impacted third molars^20^. Other studies have reported that IGF-1R is deployed within the cytoplasm of human unbilical cord blood-derived neural stem cells and localized mainly in the cytoplasm of large luteal cells^21^,^22^. These findings have promoted us to speculate that the over/low-expression of *let-7* family may modulate the expression of IGF-1R in dental stem cells. Thus, we selected the *let-7c* miRNA, one of the *let-7* family members, as the target to observe its interaction with IGF-1R in SCAPs. In the present study, IGF-1/IGF-1R/*hsa-let-7c* axis plays an important role during the committed differentiation of stem cells in which *hsa-let-7c* acts as a major modulator in IGF-1 treated SCAPs via the regulation of IGF-1R.

Meanwhile, we identified IGF-1R was localized within cytoplasm in SCAPs as the target gene of *hsa-let-7c*, and both were negatively correlated. Transfection with *hsa-let-7c* low-expression virus brought about the upregulation of IGF-1R and the enhanced odonto/osteogenic differentiation in IGF-1-treated SCAPs, as indicated by the upregulation of several odonto/osteogenic markers (*DMP1*/DMP1, *COL-I*/COL-I, ALP, *RUNX2*/RUNX2, *OSX*/OSX, *DSPP*/DSP and *OCN*/OCN) *in vitro*. On the contrary, the *hsa-let-7c* over-expression downregulated the level of IGF-1R and decreased the odonto/osteogenic differentiation in IGF-1-treated SCAPs. It is commonly believed that IGF-1 takes actions mostly via IGF-1R^23^,^24^. These findings indicate that IGF-1, IGF-1R and *hsa-let-7c* act as a whole especially in the process of cell differentiation.

Dentin-sialoprotein (DSP) is one member of the major non-collagenous proteins, which is derived from a single dentin-sialophosphoprotein (*DSPP*) gene. Both DSP protein and *DSPP* gene are highly expressed in odontoblast lineages, and considered as the typical odontogenic markers for the differentiation of human MSCs^25^,^26^. Dentin matrix protein-1 (DMP1) is expressed in odontoblasts that secrete the matrix proteins in the process of dentinogenesis and mineralization during postnatal tooth development. Some studies have suggested a potential functional and synergistical relationship between *DSPP* and *DMP1*, in which *DSPP* acts as the downstream effector molecule of *DMP1* and can be controlled by *DMP1* during the dentinogenesis and osteogenesis^27^,^28^,^29^.

Some osteogenic markers (e.g., ALP, RUNX2, OSX and COL-I) are secreted during the early stage of osteo/odontoblastic differentiation, whereas OCN is involved in the late-stage differentiation process^30^,^31^,^32^,^33^. ALP is expressed and secreted from a proliferation phase to the deposition of mature extracellular matrix including calcium phosphate during the cell differentiation^34^. *RUNX2* and *OSX* are expressed in osteogenic mesenchyme in all stages of craniofacial bone development, and *RUNX2* can modulate bone and tooth development directly or through a *RUNX2* signaling pathway related to *OSX*^32^. COL-I comprises almost 90% of organic material during the formation of inorganic bone matrix to support the structure of osteogenesis^33^,^35^,^36^. OCN is a small c-carboxyglutamate protein, which is synthesized only by mature cementoblasts, osteoblasts and odontoblasts, and its function is controlled by RUNX2 in the later-stage of mineralized tissues^35^,^36^,^37^. Our previous studies have proved that IGF-1 can stimulate the odonto/osteogenic differentiation of SCAPs and PDLSCs *in vitro*^6^,^8^. In this study, all early and late phase markers of odonto/osteogenic differentiation were highly expressed in IGF-1-treated SCAPs with the low-expression of *hsa-let-7c*, but significantly downregulated in *Let-7c* overexpression group, indicating that *hsa-let-7c* was paramount to the IGF-1/IGF-1R/*hsa-let-7c* axis.

**Figure 6:**
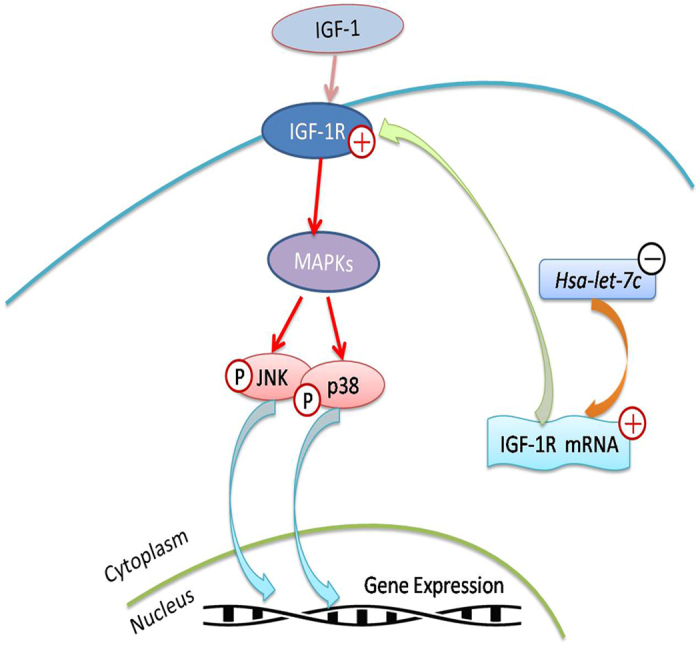
Schematic diagram for IGF-1/IGF-1R/*hsa-let-7c* MAPK axis. ERK, JNK and p38 MAPKs are members of MAPK family which can be activated by a variety of environmental factors. JNK and p38, which are activated by *hsa-let-7c* low-expression and IGF-1R over-expression, can translocate to the nucleus where they phosphorylate the transcription factors and subsequently regulate the downstream gene expression.

The differentiation potential of stem cells is subject to a variety of internal and external factors regulated by signaling pathways. Many external factors, which play a physiological role through diverse signaling pathways, can affect the biological properties of SCAPs^38^,^39^,^40^,^41^,^42^. In this study, JNK and p38 MAPK pathways were activated through *hsa-let-7c* low-expression and IGF-1R over-expression, suggesting that the activation of this pathway is associated with IGF-1/IGF-1R/*hsa-let-7c* axis (Fig. 6) during the odonto/osteogenic differentiation of SCAPs.

Mitogen-activated protein kinases (MAPKs) belong to the serine-threonine kinase family and are important component in the signal transduction networks. MAPK pathway, which is a non-classical pathway, plays a crucial role in cell growth, proliferation, differentiation and apoptosis. It mainly consists of three parallel mechanisms, i.e., extracellular signal regulated kinases (ERKs), c-Jun N-terminal kinases (JNKs) and p38 MAPK^43^. The three parallel pathways play crucial roles in regulating the odonto/osteogenic differentiation of many cell types, such as DPSCs, PDLSCs, SCAPs and MSCs^8^,^9^,^38^,^44^.

Different stimuli can activate different MAPK pathway members, and have different downstream targets. *Let-7* can mediate cell growth and differentiation via the activation of MAPK signaling pathway^45^. In detail, *let-7* can regulate the ERK MAPK and Akt/PI3K signaling pathways by targeting the estrogen receptor (ER)-α36^17^. In this study, our findings demonstrated that *hsa-let-7c* can control the activity of JNK and p38 MAPK pathways by targeting the IGF-1R. As a target of *let-7* family, IGF-1R can promote the cell proliferation through the crosstalk between IRS-2/Akt and MAPK pathways^46^,^47^. The activated IGF-1R can combine corresponding molecules to trigger the downstream signaling cascades including MAPK pathway, thereby regulating the cell transformation, growth and survival^48^,^49^. In particular, IGF-1R and p38 MAPK pathway can control the quiescence of hDPSCs and will be inactivated when DPSCs mitosis occurs^50^. As the upstream regulators of MAPK pathway, IGF-1R and *let-7* work closely as a whole in cell differentiation. Furthermore, ERK MAPK pathway can negatively regulate the expression of *let-7* via LIN28 expression induced by Myc transcription^51^. ERK MAPK signaling pathway can modulate the phosphorylation of miRNA to generate the complex, thereby affects the regulation of cell mitosis signals.

Taken together, we have a general knowledge of the relationship among three members in the IGF-1/IGF-1R/*hsa-let-7c* axis (Fig. 6). As shown in our study, IGF-1, IGF-1R and *hsa-let-7c* were tightly associated with the odonto/osteogenic differentiation of SCAPs, while IGF-1R and *hsa-let-7c* were negatively correlated. IGF-1 takes actions via binding to IGF-1R, and the latter itself can also regulate the stem cell differentiation. Moreover, *hsa-let-7c* can control cell differentiation induced by IGF-1 and IGF-1R through modulating IGF-1R expression.

IGF-1/IGF-1R/*hsa-let-7c* axis has a key influence on the odonto/osteogenic differentiation of IGF-1-treated SCAPs as well as MAPK signaling pathway. These findings indicate that we can take advantage of the modulation IGF-1/IGF-1R/*hsa-let-7c* axis on the odonto/osteogenic differentiation for tooth regeneration in the future. Further study needs to be performed to explore the downstream signals of MAPK pathway, especially changes in the level of transcription factors.

## Methods

### *Hsa-let-7c* target genes prediction

Prediction and analysis of target genes of *hsa-let-7c* were performed using bioinformatics methods. The miRDB is an online database for miRNA target prediction. And the whole targets in miRDB are predicted by a bioinformatics tool. The miRDB can predict miRNA targets in five species, including human. In this study, we utilized the miRDB database to perform the bioinformational analysis of target genes of *hsa-let-7c.*

### Cell isolation and identification

Impacted third molars (n = 24) were collected from young patients (17–20 years old) in Oral Surgery Department of Jiangsu Provincial Stomatological Hospital after the informed consent from patients was obtained. This study was approved by the Ethical Committee of the Stomatological School of Nanjing Medical University (Reference #200900128). All procedures were carried out according to the Human Care Guidelines of the Ethical Committee of Nanjing Medical University. The apical papillae were gently detached from the immature roots, minced and digested in a solution containing 3 mg/mL collagenase type I (Sigma, St. Louis, MO) and 4 mg/mL dispase (Sigma, St. Louis, MO) for 30 min at 37 °C. Then, these cells were purified by using rabbit anti-STRO-1 antibody (Santa Cruz, Delaware, CA) and sheep anti-rabbit IgG Dynabeads (Dynal Biotech, Oslo, Norway) according to the standard procedures for magnetic activated cell sorting (MACS). Purified stem cells from apical papilla (SCAPs) were seeded at 1 × 10^4^ cells/mL into 10 cm culture dishes and cultured in alpha minimum essential medium (α-MEM, Gibco, Life Technologies, Grand Island, NY) supplemented with 6% fetal bovine serum (Gibco, USA), 100 U/mL penicillin and 100 μg/mL streptomycin at 37 °C in 5% CO_2_. The fresh medium was changed every 2 days. Cells were subcultured at the ratio of 1:3 when they reached 75–85% confluence. SCAPs at 1–3 passages were used for subsequent experiments. The expression of IGF-1R in purified SCAPs was detected by the confocal laser scanning microscopy (CLSM) method (IGF-1R antibody was purchased from Boster Technology, China). Then, these stem cells were cultured in α-MEM containing 100 ng/mL IGF-1 (Peprotech, USA)^6^,^8^. Stem cells were routinely examined under the phase-contrast inverted microscope (Olympus). Generally, SCAPs at the same passage were used in each experiment.

### Selection of the best MOI value

Multiplicity of infection (MOI) refers to the average number of viral particles in a cell with an active viral infection. The best MOI value means that viral particles exert the least impact on the growth morphology of the cells, but can obtain the highest transfection efficiency. In the present study, SCAPs after transfection were plated in triplicate in 8 wells of 96-well plates at 5 × 10^4^ cells per well, and divided into eight groups as described in Table 2. 10 μL titer virus was added to the respective wells except control group. 12 hours later, virus was replaced with the conventional medium and cells were continuously cultured for 4 days to observe the growth features and fluorescence expression under an inverted microscope. Finally, MOI was calculated according to the photography records.

### Virus transfection

IGF-1 treated SCAPs were transfected with *hsa-let-7c* over/low-expression lentiviruses (Shanghai Genechem Co., LTD, Shanghai, China) and thus four groups were applied in this study, i.e., Con-over group (cells transfected with over-expression control vectors), *Let-7c*-over group (cells transfected with *hsa-let-7c* over-expression lentivirus), Con-low group (cells transfected with scramble control vectors) and *Let-7c*-low group (cells transfected with *hsa-let-7c* low-expression lentivirus). In the preliminary experiment, the best parameter of MOI was screened and its value is equal to 5. SCAPs were seeded in 25 cm^2^ culture dishes, and the culture medium was replaced after 24 hours. At the confluence of 60–70%, cells were transfected with the *hsa-let-7c* over/low-expression lentiviral vectors in 2 mL α-MEM medium containing 6% fetal bovine serum and 8 μg/mL polybrene (POL). 10 hours later, virus media were replaced with 3 mL conventional media for further culture and screening.

### Cell counting kit 8 (CCK8) assay

Transfected SCAPs were plated in triplicate in a 96-well plate at 2 × 10^3^ cells per well and incubated in the absence or presence of IGF-1. The proliferation features were determined by using the cell counting kit-8 (CCK8, Dojindo, Japan) at different time points (days 0, 1, 3, 5, 7, 9, 11, 13, respectively). After SCAPs were treated with CCK8 at 37 °C for 2 hours, the optical density was measured by microplate reader scanning at 490 nm according to the manufacturer’s instruction^52^.

### Flow cytometry

For cell cycle analysis, 5 × 10^5^ cells were harvested by trypsinization (Beyotime, China), washed twice with PBS, and then fixed with 75% ice-cold ethanol for 24 hours at 4 °C. The fixed cells were then stained with propidium iodide (PI) for 30 min at 37 °C. Finally, all samples were analyzed by flow cytometry. Cell cycle analysis was measured by FACScan flow cytometer (BD Biosciences, San Jose, CA, USA). The experiment was performed in triplicate.

### Alizarin red staining

Transfected SCAPs in Con-over group, *Let-7c*-over group, Con-low group and *Let-7c*-low group were respectively seeded into 24-well plates (Nunc, USA) at a density of 1 × 10^4^ cells/well and cultured in routine media or mineralization-inducing media (MM) (containing α-MEM, 6% fetal bovine serum, 100 U/mL penicillin, 100 μg/mL streptomycin, 100 μM ascorbic acid, 2 mM 2-glycerophosphate and 10 nM dexamethasone). At day 14, alizarin red staining was carried out as described before^38^,^53^,^54^,^55^ and images were acquired using a scanner. Then, nodule staining was destained by 10% cetylpyridinium chloride (CPC) in 10 mM sodium phosphate for 30 minutes at room temperature. The calcium concentration was determined by measuring the absorbance at 526 nm with a universal microplate reader (BioTek Instruments, USA). This experiment was performed in triplicate and the results were presented as the means ± SD.

### Real-time reverse transcription polymerase chain reaction

Total cell RNA was extracted by using TRIzol reagent (Invitrogen, New York, NY, USA) according to the manufacturer’s protocols. The concentration and purity of RNA samples were determined by the absorbance of RNA at 230, 260, and 280 nm, respectively. The mRNA was reverse-transcribed into cDNA by using a PrimeScript RT Master Mix kit (TaKaRa Biotechnology, China). Real-time RT-PCR was performed using SYBR Green Master (Roche, Indianapolis, IN, USA) and ABI 7300 real-time PCR system. Real-time RT-PCR reaction conditions for *hsa-let-7c* were: 95 °C for 10 minutes; followed by 40 cycles of 95 °C for 15 seconds, 60 °C for 60 seconds; then 72 °C for 45 seconds, 72 °C for 7 minutes, 4 °C for 5 minutes. U6 was used as an internal control and the expression of *hsa-let-7c* was measured by the method of 2^−∆∆Ct^ as previously reported^38^,^56^. Data were described as the means ± SD of three independent experiments. Real-time RT-PCR reaction conditions for dentinsialophosphoprotein (*DSPP*), dentin matrix protein 1 (*DMP1*), Runt-related transcription factor 2 (*RUNX2*), osterix (*OSX*), type I collagen (*COL-I*) and osteocalcin (*OCN*) were: 95 °C for 10 minutes; followed by 40 cycles of 95 °C for 15 seconds, 60 °C for 60 seconds. Primers used in this experiment were listed in Table 1. GAPDH was used as an internal control and the expression of osteo/odontoblast-related genes (*DSPP, DMP1, RUNX2, OSX, COL-I* and *OCN*) was measured by the method of 2^−∆∆Ct^ as previously reported^38^,^56^. Data were described as the means ± SD of three independent experiments.

### Western blot analysis

To investigate the effects of the over-expression/low-expression of *let-7c* on the odonto/osteogenic differentiation of IGF-1 treated SCAPs, IGF-1 treated SCAPs in Con-over group, *Let-7c*-over group, Con-low group and *Let-7c*-low group were respectively cultured for 0, 3 and 7 days and then collected. For the evaluation of MAPK pathway-related proteins, IGF-1 treated SCAPs at 80–90% confluence were collected after *let-7c* transfection for 0.5 hour, 1 hour and 6 hours. Cells in different groups were washed twice with cold PBS and lysed in RIPA lysis buffer (Beyotime, China) containing 1 mM PMSF. Cell debris was eliminated by centrifugation at 12,000 rpm for 10 minutes. The cytoplasm protein and nucleoprotein were obtained with an NE-PER Nuclear and Cytoplasmic Extraction Reagents (Thermo, USA). Protein concentration was measured by Bradford protein assay. Thirty microgram protein per lane was loaded onto a 10% SDS-PAGE gel for electrophoresis, and then transferred to 0.22 μm PVDF membranes (Millipore, Bedford, MA) at 300 mA for 1 hour. Membranes were blocked in blocking solution (5% Bovine Serum Albumin, 0.01 M TBS, 0.1% Tween-20) at room temperature for 2 hours, and incubated with primary antibodies (DMP1, 1:1000, Abcam; DSP, 1:1000, Santa Cruz; RUNX2, 1:1000, Abcam; OSX, 1:1000, Abcam; OCN, 1:1000, Millipore; ERK1/2, 1:1000, Bioworld; phosphor-ERK1/2, 1:1000, Bioworld; JNK1/2/3, 1:1000, Bioworld; phosphor-JNK1/2/3, 1:1000, Bioworld; p38, 1:1000, Bioworld; phosphor-p38, 1:1000, Bioworld; IGF-1Rα, 1:1000, Abcam; β-ACTIN, 1:1000, Bioworld) overnight at 4 °C. β-ACTIN was used as the internal control. Finally, the membranes were washed with TBST for 10 minutes × 3 followed by incubation in secondary antibodies (1:10,000, Boster) for 1 hour at 37 °C, visualized with ImageQuant LAS4000 system (GE Healthcare, USA). The results were quantified with Image J software (National Institutes of Health, USA). The experiment was repeated three times.

### Statistics

Two-sample *t* test and Chi-square test were respectively performed to compare the means and constituent ratios between two groups. For multiple comparisons between experimental groups and control groups, Dunnett’s test was used to check the significant differences. Two-tailed P-values less than 0.05 were considered statistically significant. All statistical analysis was performed with SPSS 17.0 software (SPSS Inc., Chicago, IL, USA).

## Additional Information

**How to cite this article**: Ma, S. *et al*. IGF-1/IGF-1R/*hsa-let-7c* axis regulates the committed differentiation of stem cells from apical papilla. *Sci. Rep.* **6**, 36922; doi: 10.1038/srep36922 (2016).

**Publisher’s note:** Springer Nature remains neutral with regard to jurisdictional claims in published maps and institutional affiliations.

## Figures and Tables

**Table 1:** Sense and antisense primers for real-time reverse transcription polymerase chain reaction.

Genes	Sequences (5′-3′)
*COL-I*	F: GAGCTGGCTACTTCTCGC
	R: TCTATCCGCATAGGACTGAC
*RUNX2*	F: AATGCCTCCGCTGTTATG
	R: TTCTGTCTGTGCCTTCTTG
*OSX*	F: GCCTACTTACCCGTCTGACTTT
	R: GCCCACTATTGCCAACTGC
*OCN*	F: AAGCCCAGCGACTCTGAGTCT
	R: CCGGAGTCTATTCACCACCTTACT
*DSPP*	F: CGTTCAGGGAGTCCTAGCGGGAACG
	R: GTGACTCTCCCTTCCATCTCCTG
*DMP1*	F: TTTTAGGAAGTCTCGCATCT
	R: TGGGACCATCTACGTTTT
*GAPDH*	F: GCCTCGTCTCATAGACAAGATGGT
	R: GAAGGCAGCCCTGGTAACC
*Hsa-let-7c*	F: ACACTCCAGCTGGGTGAGGTAGTAGGTTGT
	R: TGGTGTCGTGGAGTCG
*U6*	F: CTCGCTTCGGCAGCACA
	R: AACGCTTCACGAATTTGCGT

**Table 2:** Virus titers for multiplicity of infection (MOI) in eight groups.

Virus titers	+Conventional medium	+Conventional medium+ POL
1 × 10^7^ TU/mL virus	1	2
5 × 10^6^ TU/mL virus	3	4
1 × 10^6^ TU/mL virus	5	6
Blank control group	7	8
